# How can tuberculosis services better support patients with a diabetes co-morbidity? A mixed methods study in the Philippines

**DOI:** 10.1186/s12913-023-10015-7

**Published:** 2023-09-25

**Authors:** Lauren Oliveira Hashiguchi, Sharon E. Cox, Tansy Edwards, Mary C. Castro, Mishal Khan, Marco Liverani

**Affiliations:** 1 National Institute of Nursing Research, National Institutes of Health, 31 Center Drive, Bethesda, MD 20892-2178 USA; 2 Faculty of Public Health and Policy, London School of Hygiene & Tropical Medicine, Keppel St, London, WC1E 7HT UK; 3 School of Tropical Medicine & Global Health, Nagasaki University, 1 Chome-12-4 Sakamoto, Nagasaki, 852-8523 Japan; 4 Faculty of Epidemiology & Population Health, London School of Hygiene & Tropical Medicine, Keppel St, London, WC1E 7HT UK; 5 United Kingdom Health Security Agency, 61 Colindale Avenue London NW9 5EQ, Collindale, UK; 6 MRC International Statistics and Epidemiology Group, London School of Hygiene & Tropical Medicine, Keppel St, London, WC1E 7HT UK; 7 Nutrition Center Philippines, Muntinlupa City, Manila Philippines; 8 Aga Khan University, National Stadium Road, Karachi, 74800 Pakistan

**Keywords:** Diabetes, Tuberculosis, TB-DM, Co-morbidity, Integrated care, Self-care, Philippines, Southeast Asia

## Abstract

**Background:**

People with diabetes mellitus (DM) have an estimated two- to three-times greater risk of adverse tuberculosis (TB) treatment outcomes compared to those without DM. Blood glucose control is a primary aim of managing DM during TB treatment, yet TB programmes are not generally adapted to provide DM services. The purpose of this study was to understand perceptions and the lived experiences of diabetic patients in TB treatment in the Philippines, with a view to informing the development of disease co-management strategies.

**Methods:**

This mixed methods study was conducted within a prospective cohort of adults newly-starting treatment for drug-sensitive and drug-resistant TB at 13 public TB clinics in three regions of the Philippines. Within the subset of 189 diabetic persons who self-reported a prior DM diagnosis, or were diagnosed by screenings conducted through the TB clinic, longitudinal blood glucose data were used to ascertain individuals’ glycaemic control (controlled or uncontrolled). Univariable logistic regression analyses exploring associations between uncontrolled glycaemia and demographic and clinical factors informed purposive sampling of 31 people to participate in semi-structured interviews. All audio-recorded data were transcribed and thematic analysis performed.

**Results:**

Participants — both with controlled and uncontrolled blood glucose — were knowledgeable about diabetes and its management. However, a minority of participants were aware of the impact of DM on TB treatment and outcomes. Many participants newly-diagnosed with DM at enrolment in TB treatment had not perceived any diabetic symptoms prior and would have likely not sought clinical consult otherwise. Access to free glucose-lowering medications through TB clinics was a key enabling resource. However, participants expressed fear of side effects and interrupted access to glucose-lowering medications, and a preference for phytotherapy. Many participants felt that physical and financial impacts of TB and its treatment were challenges to DM management.

**Conclusions and recommendations:**

Results of this study indicate that public TB clinics can provide diabetic patients with additional health care resources and education to address co-morbidity. TB programmes might consider identifying patients with complicated DM, and offering diabetic monitoring and management, as DM and diabetic complications may compound the burden of TB and its treatment.

**Supplementary Information:**

The online version contains supplementary material available at 10.1186/s12913-023-10015-7.

## Background

People with diabetes mellitus (DM) have an estimated two- to three-times greater risk of poor tuberculosis (TB) treatment outcomes compared to those without DM [1–9]. Improving the management of DM among persons with TB could optimise TB treatment outcomes [10–14]. Long-term hyperglycaemia not only contributes to complications of DM, such as nerve and kidney damage, but likely has a role in affecting TB treatment outcomes [15, 16]. Given the heighted risk of adverse TB treatment outcomes among patients with a DM comorbidity (TB-DM), blood glucose control is an especially important part of diabetic management.

Glycaemic control is influenced by clinical intervention and self-care behaviours, such as healthy eating, being physically active, self-monitoring of blood glucose and using prescribed glucose-lowering medications [17–19]. Non-adherence to recommended self-care behaviours—often driven by limited access to diabetic support services [18]—contributes to poor glycaemic control [20–22]. Among persons with DM, adherence to self-care practices is affected by factors such as knowledge about diabetes, self-efficacy, depression, medical beliefs, medical cost and social support [18, 23, 24].

Maintaining diabetic self-care practices is particularly challenging in the context of multiple comorbidities [25, 26], especially TB which can cause physical and economic hardship and requires long-term, daily observed treatment [27]. However, TB and diabetes are generally treated in separate facilities and their integration is particularly challenging in low- and middle-income country (LMIC) settings due to more limited health sector resources and the organisation of health service delivery in vertical structures [28]. This introduces the potential for conflicting clinical management [29] and additional risks as TB patients defer care or seek care at DM or general clinics where they might transmit TB [30].

The TB Directly Observed Treatment Strategy (TB-DOTS) framework could be adapted to support diabetic management to reduce the risk of poor TB treatment outcomes and risk of diabetes-related complications [12], but there is limited evidence to guide how services should be organised. Thus, understanding factors which influence DM self-care from the perspective of persons in TB treatment is critical to developing co-management strategies.

The setting of this study is the Philippines, a lower-middle income country in Southeast Asia which has a high TB incidence (539/100,000), compared to the WHO region of Southeast Asia (234/100,000) [31]. In 2020, the International Diabetes Federation estimated that DM affects 6.5% of Filipino adults [32]. No studies have assessed barriers to diabetic management among TB-DM patients in in Southeast Asia, and there is a lack of evidence exploring barriers to diabetic self-care in the context of any comorbidity in LMICs. We therefore sought to understand perceptions and the lived experiences of diabetic patients in TB treatment in the Philippines, with a view to informing the development of disease co-management strategies.

## Methods

This study adheres to the Consolidated Criteria for Reporting Qualitative Research (COREQ) recommendations (Additional file 1) [33].

### Design 

This explanatory, sequential mixed methods study explored challenges to diabetic self-care and the co-management of TB and diabetes [34, 35]. First, longitudinal glycosylated haemoglobin (HbA1c) data, a test which reflects average glycaemic control over one to three months, were used to assess overall trends in glycaemic control during TB treatment at public facilities among a cohort of patients with diabetes. Univariable logistic regression analyses were used to identify associations with having controlled versus uncontrolled glycaemia, and results informed sampling in the subsequent qualitative phase. Between June and August 2020, we conducted semi-structured interviews with participants with diabetes receiving treatment for TB though public facilities to understand patient-level experiences managing their diabetes while receiving treatment for TB.

### Setting

This study was conducted within the Starting Anti-TB Treatment (St-ATT) prospective cohort study of 901 non-pregnant adults (≥ 18 years) initiating TB treatment for both drug-sensitive and drug-resistant TB infection in public TB-DOTS clinics in the Philippines (ISRCTN16347615) [36]. St-ATT sites included 13 public TB-DOTS clinics in Metro Manila (*N* = 3), Cebu (*N* = 5) and Negros Occidental (*N* = 5). Four sites were centres for the programmatic management of drug resistant TB (PMDT). Sites in Cebu and Negros Occidental were in urban, peri-urban and rural areas, and sites in Manila were in urban areas. In the St-ATT study, DM cases are presumed Type 2, which typically develops in adulthood and is considered a lifestyle-related disease.

### Theoretical framework

**Fig. 1:**
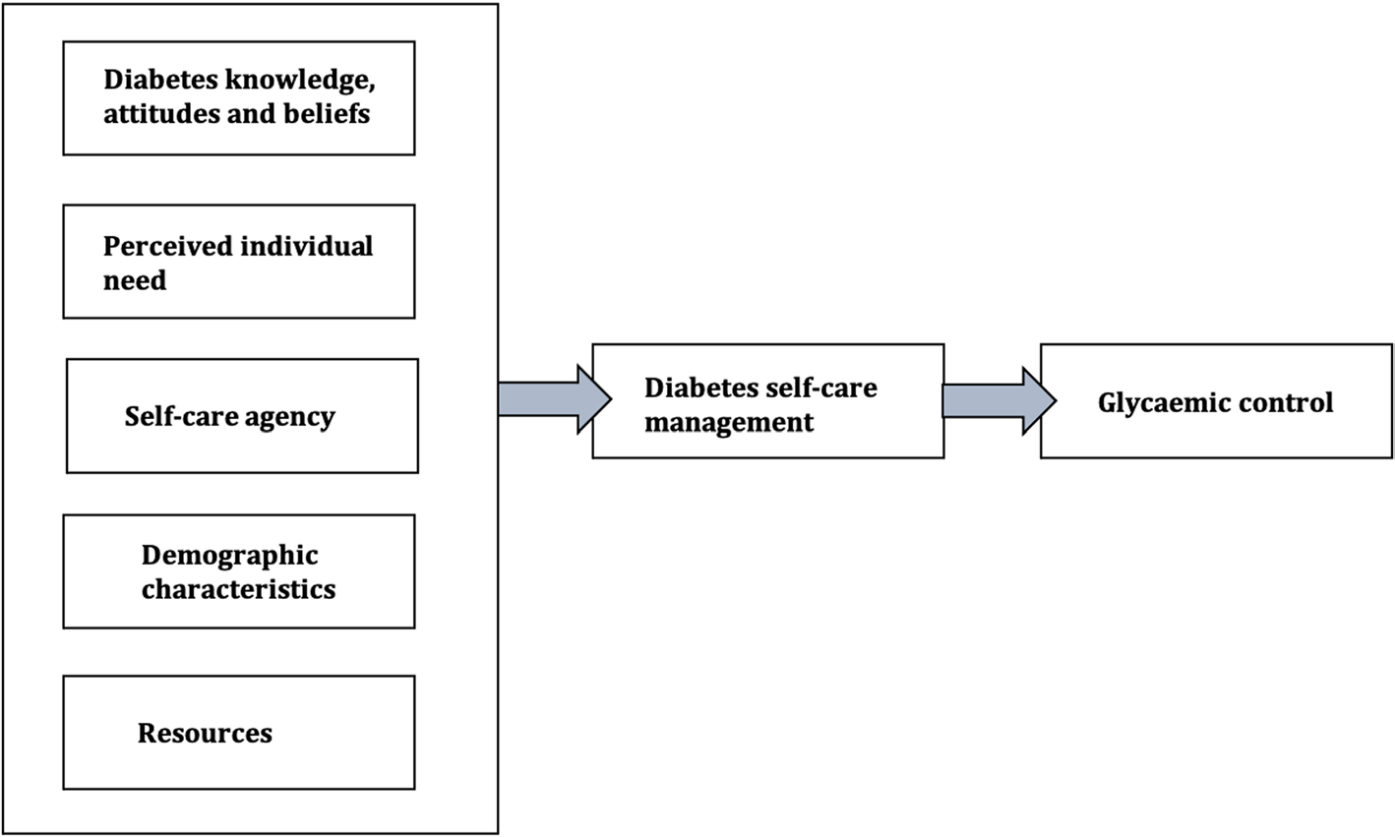
Adapted Theory of Diabetes Self-Care Management

The study framework (Fig. 1) adapted Sousa and Zauszniewski’s Theory of Diabetes Self-Care Management (TDSCM) [19, 37, 38] and Melkamu et al.’s (2021) diabetes-adapted Health Belief Model [39]. It proposes that demographic characteristics (e.g., age, sex or duration of disease), knowledge and attitudes about DM and its management, access to resources (i.e., material resources or social support), agency (i.e., the ability to plan and execute self-care activities), and how an individual perceives the severity of their diabetic condition influence DM self-care management behaviours [40, 41].

### Recruitment and sample

We recruited from the 189 St-ATT participants who either self-reported a prior DM diagnosis, or were diagnosed by DM screenings conducted by the St-ATT study between June and August 2020 using purposive sampling techniques. At enrolment and trimonthly, the St-ATT study measured HbA1c. HbA1c data were used to categorise participants into glycaemic control outcomes: controlled, uncontrolled, or indeterminate.^1^ Within each of the glycaemic control outcomes, we aimed to achieve maximum variation of key characteristics measured in St-ATT surveys, identified by univariable logistic regressions which estimated the odds of having controlled versus uncontrolled glycaemia among St-ATT participants with TB-DM (Wald test *P* < 0.05, Table 1).

**Table 1:** Univariable Associations with Uncontrolled Blood Glucose among 189 TB patients with diabetes comorbidity

Characteristic	Total	Uncontrolled^a^ (%)	Odds Ratio (95% CI)	Wald *P*-value
Age (years)	151	66 (43.7)	1.00 (0.97, 1.03)	0.993
Sex				0.083
Female	46	25 (54.3)	1	
Male	105	41 (39.0)	0.54 (0.27, 1.08)	
Region				0.012
Cebu	70	27 (38.6)	1	
Negros Occidental	66	26 (39.4)	1.04 (0.52, 2.06)	
Manila	15	13 (86.7)	10.35 (2.17, 49.49)	
Absolute household income				0.754
Less than 5,000 PHP	63	29 (46.0)	1	
5000, 9999 PHP	39	15 (38.5)	0.73 (0.32, 1.65)	
> 10,000 PHP	48	21 (43.8)	0.91 (0.43, 1.94)	
Marital status				0.127
Single	29	10 (34.5)	1	
Married	109	53 (48.6)	1.80 (0.77, 4.22)	
Divorced, separated or widowed	13	3 (23.1)	0.57 (0.13, 2.56)	
Employment status				0.919
Unemployed	100	44 (44.0)	1	
Employed	51	22 (43.1)	0.97 (0.49, 1.91)	
Insurance (public or private)				0.523
No insurance	45	18 (40.0)	1	
Insurance	94	43 (45.7)	1.26 (0.61, 2.60)	
BMI classification at baseline^b^				0.016
Normal (18.5–25.0 kg/m^2^)	91	42 (46.2)	1	
Underweight (< 18.5 kg/m^2^)	36	9 (25.0)	0.39 (0.16, 0.92)	
Overweight or obese (≥ 25.0 kg/m^2^)	24	15 (62.5)	1.94 (0.77, 4.90)	
Blood pressure at baseline^c^				0.083
Normal (SBP < 120 mmHg, DBP < 80 mmHg)	61	22 (36.1)	1	
Elevated (SBP 120–129 mmHg, DBP < 80 mmHg)	12	3 (25.0)	0.59 (0.14, 2.41)	
Stage 1 Hypertension (SBP 130–139 mmHg, DBP 80–89 mmHg)	43	25 (58.1)	2.46 (1.11, 5.48)	
Stage 2 hypertension (SBP ≥ 140 mmHg, DBP ≥ 90 mmHg)	19	9 (47.4)	1.60 (0.56, 4.52)	
Central obesity^d^				0.001
No	49	12 (24.5)	1	
Yes	102	54 (52.9)	3.47 (1.62, 7.41)	
Timing of DM diagnosis^e^				< 0.001
Newly diagnosed	90	26 (28.9)	1	
Previously diagnosed	61	40 (65.6)	4.69 (2.33, 9.42)	
Use of metformin^f^				< 0.001
No use	58	12 (20.7)	1	
Use	93	54 (58.1)	5.31 (2.49, 11.32)	
Use of insulin^f^				0.008
No use	132	52 (39.4)	1	
Use	19	14 (73.7)	4.31 (1.46, 12.67)	
Experience of DM complication^g^				0.048
Never	52	8 (28.6)	1	
Yes	134	58 (47.2)	2.23 (0.91- 5.45)	
New versus relapse TB case				0.029
New	96	48 (50.0)	1	
Relapse	54	17 (31.5)	0.46 (0.23, 0.93)	
Type of TB infection				0.083
Drug sensitive	118	56 (47.5)	1	
Drug resistant	33	10 (30.3)	0.48 (0.21, 1.10)	

In addition to basic sociodemographic variables, results for univariable regressions with a Wald *P*-value < 0.1 shown

*BMI* Body Mass Index, *DBP* Diastolic Blood Pressure, *DSSM* Direct sputum smear microscopy, *HbA1c* Glycosylated haemoglobin, *HT* Hypertension, *mmHg* Millimetre of Mercury, *PHP* Philippine peso, *SD* Standard deviation, *SBP* Systolic blood pressure, *TB* Tuberculosis

^a^Among study participants with ≥ 2 HbA1c results: uncontrolled (at least two study-measured HbA1c results equal to or greater than 8%); controlled (at least two study-measured HbA1c results less than 8%). Status was indeterminate if HbA1c data did not qualify into a glycaemic control category. 38 of the 189 patients had an indeterminate status and were not included in regression analyses

^b^World Health Organization (WHO) criteria for adults [42]

^c^2017 American College of Cardiology and American Heart Association guidelines [43]

^d^WHO recommendations for diagnosing metabolic syndrome [44]: > 0.85 for women and > 0.9 for men

^e^Previously-diagnosed: self-report of doctor diagnosis at enrolment into TB treatment, confirmed by self-reported use of standard glucose-lowering medication (insulin, gliclazide, metformin) if baseline HbA1c < 6.5%. Newly-diagnosed: HbA1c ≥ 6.5% test result during their TB treatment period, confirmed by self-report of an outside DM diagnosis or use of a standard glucose-lowering if no subsequent HbA1c ≥ 6.5%

^f^Self-reported any point during TB treatment

^g^After enrolment in TB treatment, report of experiencing diabetic complications [45]: ever lost a limb or digit not through trauma, ever had a bypass or stenting surgery in limbs, non-healing wound for three or more months, heart attack, stroke, bypass or stenting heart surgery, diagnosis of angina or heart failure, cataract or laser eye surgery, glaucoma, acquired blindness not due to trauma, difficulty seeing or disturbed vision, renal failure, and if participant had any symptom of distal symmetrical peripheral neuropathy using the Michigan Neuropathy Screening Instrument [46]

Key characteristics included whether participants: were diagnosed with DM prior to or after their current TB diagnosis; overweight (determined by body mass index) or with central obesity (determined by waist-hip-ratio); self-reported use of glucose-lowering medications or experience of diabetic complications; or had a relapse case of TB. We aimed to recruit an initial 5–6 participants within each glycaemic control group, continuing until data saturation was reached. Data saturation was defined as the point at which emerging issues were clear and no additional barriers or enablers to DM management were identified.

### Data collection

Interview questions were pre-defined to address the aims of the study (Supplementary File 2). Interviews were conducted with informed consent in local languages (Tagalog, Cebuano, Hiligaynon, or English) by one of two trained research assistants. One research assistant was male, the other female. Both were in their 50 s with post-graduate education in social sciences in the Philippines, and prior experience conducting qualitative interviews. Interviews were either conducted in-person at the TB-DOTS clinic, or at the participants’ home if requested, lasting 45 min to an hour. When COVID-19 lockdowns were in place, interviews were conducted by phone, lasting 30 to 45 min. Two participants had hearing loss and consented to have their interview undertaken with assistance from a family member, who listened to and repeated questions.

### Data analysis 

All interviews were recorded and transcribed verbatim. Prior to analysis, transcripts of interviews conducted in Tagalog, Cebuano, Hiligaynon were translated into English by local translators and verified for accuracy by the research assistant who conducted the interview. Data were analysed using a reflective thematic approach [47]. In the first step of analysis, the principal investigator (PI) read and re-read the initial 15 transcripts and field notes from in-person interviews to familiarize with the data. Selective coding based on framework domains (e.g. knowledge and attitudes, resources, etc.) was performed. Then, open coding was performed to enable the identification of emerging themes within the domains, and other issues which may not have been accounted for in initial research design. Themes were shared with the co-authors, with additional refinement of the coding until no new concepts were identified. Finally, codes were organised into themes, and analysed across and within each glycaemic group. Data were managed and coded using QSR NVivo12 (released in July 2021) [48].

## Results 

Participant characteristics (*N* = 31) demographic and disease characteristics are described in Table 2. The median age was 52 years (range 30–83) and most were male (*N* = 22). More than half were being treated for drug-sensitive TB (*N* = 20), and had been diagnosed with DM prior to TB treatment (*N* = 18). Twelve participants (38%) had controlled glycaemia, 14 (45%) had uncontrolled glycaemia, and five had indeterminate control (16%).

**Table 2:** Socio-demographic, anthropometric, TB- and DM-related characteristics of study participants by glycaemic control outcome

	All (*N* = 31)	Controlled^a^ (*N* = 12)	Uncontrolled^a^ (*N* = 14)	Indeterminate (*N* = 5)
Age, years				
Mean, SD	52.0 (12.4)	48.2 (11.3)	60.6 (15.1)	52.0 (12.4)
Median, Range	52.0 (30.0—83.0)	54.5 (30.0—69.0)	47.0 (31.0—66.0)	63.0 (43.0—83.0)
Sex				
Female	9	2 (22%)	5 (56%)	2 (22%)
Male	22	10 (45%)	9 (41%)	3 (14%)
Region				
Negros Occidental	13	6 (46%)	6 (46%)	1 (8%)
Cebu	10	5 (50%)	4 (40%)	1 (10%)
Manila	8	1 (12%)	4 (50%)	3 (38%)
Income level				
Less than 5,000 PHP	13	6 (46%)	6 (46%)	1 (8%)
5000—9999 PHP	5	1 (20%)	3 (60%)	1 (20%)
> 10,000 PHP	13	5 (38%)	5 (38%)	3 (23%)
Marital status				
Single	4	1 (25%)	2 (50%)	1 (25%)
Married	21	8 (38%)	11 (52%)	2 (10%)
Divorced/separated	2	0	1 (50%)	1 (50%)
Widowed	4	3 (75%)	0	1 (25%)
Unemployed				
Yes	18	7 (39%)	9 (50%)	2 (11%)
No	13	5 (38%)	5 (38%)	3 (23%)
Insurance (public or private)				
No insurance	6	3 (50%)	2 (33%)	1 (17%)
Insurance	21	7 (33%)	10 (48%)	4 (19%)
BMI classification^b^				
Normal (18.5–25.0 kg/m^2^)	22	10 (45%)	10 (45%)	2 (9%)
Underweight (< 18.5 kg/m^2^)	5	2 (40%)	2 (40%)	1 (20%)
Overweight or obese (≥ 25.0 kg/m^2^)	4	0	2 (50%)	2 (50%)
Blood pressure at baseline^c^				
Normal (SBP < 120 mmHg, DBP < 80 mmHg)	10	5 (50%)	5 (50%)	0
Elevated (SBP 120–129 mmHg, DBP < 80 mmHg)	2	2 (67%)	1 (33%)	0
Stage 1 Hypertension (SBP 130–139 mmHg, DBP 80–89 mmHg)	11	3 (30%)	6 (60%)	1 (10%)
Stage 2 Hypertension (SBP ≥ 140 mmHg, DBP ≥ 90 mmHg)	8	2 (25%)	2 (25%)	4 (50%)
Central obesity^d^				
Normal	4	3 (75%)	1 (25%)	0
Central obesity	27	9 (33%)	13 (48%)	5 (19%)
Timing of DM diagnosis^e^				
Newly diagnosed	13	6 (46%)	4 (31%)	3 (23%)
Previously diagnosed	18	6 (33%)	10 (56%)	2 (11%)
Use of insulin^f^				
No use	26	9 (35%)	12 (46%)	5 (19%)
Use	5	3 (60%)	2 (40%)	0
Use of metformin^f^				
No use	3	1 (33%)	1 (33%)	1 (33%)
Use	28	11 (39%)	13 (46%)	4 (14%)
Report of any DM complication^g^				
No	21	7 (33%)	9 (43%)	5 (24%)
Yes	10	5 (50%)	5 (50%)	0
New versus relapse TB case				
New	20	6 (30%)	11 (55%)	3 (15%)
Relapse	10	6 (60%)	2 (20%)	2 (20%)
Type of TB infection				
Drug Sensitive	20	6 (30%)	10 (50%)	4 (20%)
Drug Resistant	11	6 (55%)	4 (36%)	1 (9%)

In addition to basic sociodemographic variables, characteristics shown only for factors found to be significant in univariable regressions with a Wald *P*-value < 0.1 (Table 1)

*BMI* Body Mass Index, *DBP* Diastolic Blood Pressure, *DSSM* Direct sputum smear microscopy, *HbA1c* Glycosylated haemoglobin, *HT* Hypertension, *mmHg* Millimetre of Mercury, *PHP* Philippine peso, *SD* Standard deviation, *SBP* Systolic blood pressure, *TB* Tuberculosis

^a^Amongst those with ≥ 2 HbA1c results: Uncontrolled (at least two study-measured HbA1c results equal to or greater than 8%); controlled (at least two study-measured HbA1c results less than 8%). Status was indeterminate if HbA1c data did not qualify into a glycaemic control category

^b^World Health Organization (WHO) criteria for adults [42]

^c^2017 American College of Cardiology and American Heart Association guidelines [43]

^d^Waist-to-hip ratio using WHO recommendations for diagnosing metabolic syndrome [44]; > 0.85 for women and > 0.9 for men

^e^Previously-diagnosed: self-report of doctor diagnosis at enrolment into TB treatment, confirmed by self-reported use of standard glucose-lowering medication (insulin, gliclazide, metformin) if HbA1c result < 6.5%. Newly-diagnosed: HbA1c ≥ 6.5% test result during their TB treatment period, confirmed by self-report of an outside DM diagnosis or use of a standard glucose-lowering if no subsequent HbA1c results ≥ 6.5%

^f^Self-reported any point during TB treatment

^g^After enrolment in TB treatment, report of experiencing diabetic complications [45]: ever lost a limb or digit not through trauma, ever had a bypass or stenting surgery in limbs, non-healing wound for three or more months, heart attack, stroke, bypass or stenting heart surgery, diagnosis of angina or heart failure, cataract or laser eye surgery, glaucoma, acquired blindness not due to trauma, difficulty seeing or disturbed vision, renal failure, and if participant had any symptom of distal symmetrical peripheral neuropathy using the Michigan Neuropathy Screening Instrument [46]

The analysis identified themes within four domains of the conceptual framework (knowledge, attitudes and beliefs about DM and its management, illness perception, self-care agency, and access to resources). Themes and subthemes within these domains are discussed:

### Knowledge, attitudes, and beliefs about DM and its management 

There was general acknowledgement of DM as a serious and long-term condition which could cause complications like slow-healing wounds or vision loss. There was also general knowledge of self-care practices for DM across all glycaemic control groups, especially regarding restricting consumption of rice and sugary beverages, exercising regularly, and using glucose-lowering medications.

Several participants (*N* = 12), of which five had uncontrolled blood glucose, spoke about lowering blood glucose through phytotherapy. Phytotherapies included consuming bitter vegetables like sweet potato, guyabano, or malunggay leaves, okra, or bitter gourd, as well as foods like miracle fruit to lower blood glucose. Most participants who used glucose-lowering medications reported using phytotherapy at the same time. However, three participants spoke about switching to phytotherapy alone when they felt their blood glucose was controlled.

While many participants felt that glucose-lowering medications were effective, a minority believed they were harmful, especially in combination with anti-TB medications. Eight participants across both glycaemic control groups expressed concern that glucose-lowering or TB medications caused harm, especially damage to organs. Consequently, three participants spoke about skipping doses or stopping medication use altogether, as the following quotation illustrates:

*“When you take so many medicines, you're also worried about yourself. Of course, many people have dosages of medicine that their kidney cannot handle; there can be kidney failure because of too many medicines. I am afraid of this. That's why sometimes I don't continuously take my [diabetes] medicine. When I feel that [my blood sugar] is elevated, that is the only time I will take my maintenance medicines, for about a week. I will continue again then sometimes I would take in intervals if it seems that because my sugar is high, that my vision is blurred.”*

*(M303, Manila, Male, Uncontrolled, drug-sensitive TB)*

Due to a mistrust of glucose-lowering medications, several (*N* = 11) participants in both glycaemic control groups preferred phytotherapy, as a participant explained:

*M287: [Metformin’s] the only medicine they gave me because I was afraid to get other medicines. Isn’t it that metformin may cause something? What I would do is to maintain my diet, not drink soft drinks, not take anything with sugar, not do anything that is not good for diabetes. I stopped taking [metformin]… I don’t take metformin anymore…there should just be self-control in your diet. I also drink Guyabano. If you boil the leaves, they say it is good for diabetes.*

*(M287, Manila, Male, Controlled, Drug-sensitive TB)*

Many participants (*N* = 16) explained that they had received instructions about DM self-care— primarily regarding glucose-lowering medications and reducing the intake of certain foods— from their TB-DOTS provider, private physician, or their ST-ATT research nurse. For two participants, their St-ATT research nurse was the only source of DM health education they recalled receiving. Four participants mentioned they had not received any information about DM or its management through their TB-DOTS centre.

While there was a general trust in instructions for medication use and diet changes from their health care providers, some participants (*N* = 7) felt information from health care providers was not comprehensive or useful. For this reason, four participants explained that they sought out information from alternative sources, as illustrated by one account:

*“…I was looking for answers. I was looking for answers to my questions because like what I said earlier my doctor didn’t even explain to me what is diabetes? Is diabetes curable? Is it reversible? Is it a dietary disease? Is it a progressive disease? Like that…all of these things I have tried, I got from the internet.*

*(C512, Cebu, Male, Controlled, MDR-TB)*

When discussing their knowledge of diabetes and practice of self-care, many participants (*N* = 17) cited information gained from friends or family living with DM, or from the internet or radio. Participants relied on community sources to learn about the symptoms of diabetes, dietary practices, and potential treatments. In particular, seven participants received information about phototherapeutic remedies from family and friends.

### Illness perception

Participants generally possessed a self-awareness of their diabetes, either through clinical cues (i.e., blood tests and clinician input), or through body cues (i.e., the ability to recognise sensations in the body as a sign of glucose dysregulation). When asked to describe their DM and how they knew when their blood was elevated, fourteen participants across glycaemic control outcomes discussed using the HbA1c results received during TB treatment to assess glycaemic control. However, many participants (*N* = 12)—especially among those with uncontrolled blood glucose— had a self-awareness of their diabetes which relied on body cues. When describing the state of their diabetes, participants would speak about sensations they attributed to glycaemic dysregulation and an understanding of how their body responded to certain circumstances (e.g., when medication was used, when sugary foods were consumed):

*“With my sugar, I still don’t know now how high it is. I would feel in my body that it is high because I know what the indications are. My eyes are blurring, I feel dizzy. It’s always like that, then my hands are numb. Now my hands are stiff. Even my legs are numb. Those were the indications for me.”*

*(M303, Manila, Male, Uncontrolled, drug-sensitive TB)*

For many participants, a heightened sense of vulnerability to poor health and diabetic complications was a cue to action. More than half of study participants had been previously diagnosed with DM (18/31) (Table 2). Several of the participants (*N* = 6) with previously-diagnosed DM spoke about how the experience of acute illness (most often the onset of TB disease) compelled them to start self-care practices, whereas prior to acute illness they had not felt DM was a serious issue requiring attention. This was primarily observed among persons with uncontrolled glycaemia. For example, a participant explained that the diabetes screening received through their TB centre motivated them to begin diabetic self-care:

*“Interviewer: What happened that made you decide to get treated for diabetes?*

*N155: Because when I [got] tuberculosis, I learned that I also have diabetes, so there’s two illnesses.*

*Interviewer: But what came first, diabetes or tuberculosis?*

*N155: Diabetes first, but I didn’t take care of it. Then my tuberculosis got severe and I was referred to PMDT. After some laboratory tests, I learned my blood sugar was high, that I already have diabetes.”*

*(Male, Negros Occidental, Uncontrolled, MDR-TB)*

Four participants with uncontrolled glycaemia explained that they believed DM could cause or worsen their TB disease. For this reason, there was a feeling that diabetes management should not be neglected during TB treatment:

*“N155: [Diabetes can affect other parts of the body] like kidney, lungs, blood, heart…your whole body is affected, that’s what diabetes does. Instead of recovering, it will cause complications. You have to cure it first before other diseases.*

*Interviewer: Where did you get that information?*

*N155: I heard it from [the PMDT physician], he said to cure diabetes first, that whatever medicine you’re taking for [TB] won’t cure you if your blood sugar is not normalised.”*

*(Male, Negros Occidental, Uncontrolled, MDR-TB)*

### Self-care agency

Participants generally felt they understood the general guidelines for diet and exercise, knew how to access and use glucose-lowering medications, and were capable of self-care behaviours. The effects of TB disease, and of its treatment was found to inhibit self-care agency and negatively impact engagement in self-care. Several participants (*N* = 12) spoke about their TB and its treatment affecting their ability to engage in diabetic self-care activities. Three participants spoke about how they prioritised attention and resources to their TB treatment. For example, one participant explained:

*“This past year, I did not prioritise going to clinic for my diabetes check-up because I just focused on my lungs, and taking the medicines for TB. I also take medicines for diabetes but that's just it. I don't go for check-up anymore.*

*(M729, Manila, Female, Uncontrolled, Drug-sensitive TB)*

Participants commonly explained that the effects of TB disease and its treatment were a barrier to using glucose-lowering medications, restricting their diet, and exercising. In particular, several participants (*N* = 8) spoke about how they could not follow guidance for diet changes because they struggled with weight loss, nausea, or appetite loss from their TB infection or its treatment. For example, one participant explained:

*“Now I have metformin as my medicine, and then I'm just fixing what I eat. But sometimes, if you fix your eating, you get thinner and thinner.”*

*(N298, Negros Occidental, Male, Uncontrolled, Drug-resistant TB)*

### Access to health care resources and medication 

Twelve participants were diagnosed with DM through the initial blood sugar screening, which was offered through the St-ATT study in addition to the services of public TB-DOTS programmes. Of these, only two persons mentioned feeling any diabetic symptoms prior to their screening. Participants with newly-diagnosed DM often mentioned that they had initiated DM treatment through their TB-DOTS centre following their diagnosis.

Seven participants across glycaemic control case types stated that they exclusively sought services for DM through public facilities because services were free. This finding was observed among participants with newly-diagnosed and previously-diagnosed and treated for DM. For example, one participant with a previous DM diagnosis explained that they had stopped seeking DM services at other facilities after they learned of free services through TB-DOTS:

*“I only have consultations [at TB-DOTS]. I don’t go anywhere else for check-ups because it’s free here.”*

*(N165, Negros Occidental, Male, Controlled, MDR-TB)*

Among study participants, 28 were using metformin, and five were using insulin (Table 2). Metformin (*N* = 28) and insulin (*N* = 5) were the primary resources participants reported accessing through their TB-DOTS or health centre (Table 2). There was a general perception that accessing medicines through TB-DOTS, health centres, or barangay health stations was straightforward. Six participants mentioned medication stock-outs but all stated that this was temporary. A few participants (*N* = 4) with previously-diagnosed diabetes changed their previous prescription to a medication they could get for free through TB-DOTS, as one patient explained:

*“I also told [my TB-DOTS nurse] that I have diabetes, and that I already have the maintenance medicine [Glimepiride] for that. They said they have free metformin for diabetes and it's free here. I am trying it, but it's as if I'm not adjusted yet because I frequently have headaches.”*

*(N350, Negros Occidental, Female, Controlled, Drug-sensitive TB)*

Financial incentives (i.e., participation allowances or entitlements) were reported as a key enabler of DM self-care across glycaemic control outcomes. Participants received 250 Philippine pesos (PHP) (approximately €4.30) at each follow-up appointment for the St-ATT study, and those receiving care at PMDT clinics received a weekly allowance of 700 PHP (€12.00) conditional on their adherence to TB treatment. Five participants enrolled in treatment through PMDT centres used their weekly allowance to purchase insulin, needles, or metformin when they could not get them through PMDT. For example, one participant explained:

*“The syringes, we buy those if they have no stock. There was a time that they didn't have enough insulin, so we bought it. We used the allowance they give every week. That's what we used to buy the insulin.”*

*(N321, Negros Occidental, Female, Uncontrolled, MDR-TB)*

Though PMDT centres had enhanced resources for diabetes management, three participants at TB-DOTS facilities in Negros spoke about periods when insulin was not in stock. Two of these participants had suspended their insulin use during this time rather than purchase the medicines out-of-pocket, as illustrated by one participant’s account:

*N155: There was a time that I stopped having insulin because it’s costly, they told me if I become dependent with insulin it will be for a lifetime. You need a vial every two weeks your whole life. That’s difficult, but I can’t avoid insulin, can I?*

*Interviewer: So, what do you do when you don’t buy insulin?*

*N155: I just skip using it every now and then...*

*(Male, Negros Occidental, Uncontrolled, MDR-TB)*

Various reasons for not accessing public resources for DM management were mentioned. Five participants were not aware of free glucose-lowering medications at their TB-DOTS centre. In some cases, participants assumed diabetes management was beyond the remit of TB-DOTS, as explained by one participant:

*“Interviewer: Did they give you medicines for diabetes?*

*M729: No. But I didn't ask them yet about this since I'm just consulting there about my lungs.”*

*(Manila, Female, Controlled, Drug-sensitive TB)*

Seventeen participants entitled to free DM medication purchased it out-of-pocket for various reasons. A few participants (*N* = 5) did so to avoid travelling to and waiting at a health centre, and did not feel it was financially burdensome:

*“I just bought [metformin] with my wife because there are too many people [in the TB-DOTS centre] and if you are late you end up waiting till noon time… It’s cheap. It’s 5 pesos.”*

*(C186, Male, Cebu, Uncontrolled, Drug-sensitive TB)*

In five interviews, participants shared that glucose-lowering medications were prescribed but not available through their TB-DOTS facility, so their only option was to purchase medicines out-of-pocket. Several participants (*N* = 5) spoke about borrowing money from family to pay for glucose-lowering medications. A few people (*N* = 4) stated that their economic hardship was caused by unemployment due to their TB infection, as highlighted by a participant:

*N 11: And then, you lost your job. For eight months you don’t have income, you are dependent on your children. It’s so difficult, as if you are begging alms. It's really different spending money for your maintenance medicines when you know that you can’t support yourself. It’s so difficult. You don’t have work and now you have this sickness, it’s so difficult.*

*(M303, Manila, Male, Uncontrolled, Drug-sensitive TB)*

## Discussion

Understanding the lived experience of persons managing diabetes during TB treatment is a critical component of identifying gaps in DM services, and discovering how services might be better organised to support patients. In this study, accounts of 31 diabetic participants’ experience of managing DM during TB treatment were used to understand factors that may have impacted impact blood glucose control during treatment. Findings from this project raise questions about how services for diabetes support might be organised and targeted through TB-DOTS programmes in the Philippines, and suggest areas of focus for future research addressing the needs of people with DM across settings.

### Knowledge, attitudes, and beliefs

Previous studies found a lack of knowledge regarding DM and its management is a barrier to self-care [49–51]. This study found that subjects generally understood diabetes, its symptoms, and its management and did not consider their knowledge a barrier to management. This finding was in keeping with a previous study conducted among Cambodian persons living with DM and/or hypertension—most subjects had knowledge of their disease and its management, but did not have the resources or agency to implement self-care [52].

This study found that subjects often preferred phytotherapy over glucose-lowering medications, and that members of the community were a key source of information about phytotherapy. Participants often linked their preference of phytotherapy to concern that TB or DM medications were causing harm to internal organs. Preference of phytotherapy among participants was consistent with systematic reviews exploring barriers and enablers to DM self-management among South Asian [53] and Latin America and the Caribbean populations [23], which identified a preference for traditional medicine and phytotherapy and similar mistrust of DM medications. This finding was consistent with another systematic review focused on Latin America and the Caribbean [23].

In this study the preference for phytotherapy was not identified as a barrier or enabler of DM self-care. However, the underlying mistrust of glucose-lowering medications indicates a need for education about the value of maintaining use of these medications during TB treatment. Studies of adherence to chronic disease management indicate that improving education alone was rarely sufficient to improve adherence to self-care behaviours, including medication use [54, 55]. Therefore, while health education about DM treatment offered through TB programmes may be helpful, further supportive resources, including culturally appropriate messaging regarding the use of phytotherapies, are needed.

While knowledge of one’s blood glucose levels is not sufficient to improve self-efficacy and motivate patients to engage in self-care, it is supportive [56]. Self-monitoring of blood glucose may be associated with improved HbA1c levels among persons with DM [57, 58]. In this study, participants discussed their diabetes using the blood glucose readings received through the St-ATT study, but few spoke about having their blood glucose tested outside of TB-DOTS or considered self-monitoring of blood glucose part of self-care activities. This is consistent with study survey data: nearly half (*N* = 13/31, Table 2) of participants in this study were diagnosed with diabetes through screening offered at their TB treatment centre and would not have yet needed to seek other services for routine blood glucose monitoring. Through the Philippine Package of Essential Non-Communicable Disease Intervention (PhilPEN) and primary care benefits package (Enhanced Primary Care Package), all public health centres should offer standardised management of DM, including blood glucose monitoring and provision of free maintenance medications for DM. In our study sites, participants reported that these resources were not always available. Increasing access to free blood glucose monitoring through health centres, and linking services to TB-DOTS may be an opportunity to enhance patient knowledge of their disease and support self-care activities. However, health authorities managing the local health system should monitor how accessible and responsive DM services offered through public health facilities, including TB treatment centers, are.

### Illness perception

Perceptions of illness—how a person cognitively appraises and personally understands a medical condition and its potential consequences [59, 60]—can influence engagement in self-care [61, 62]. A 2011 meta-analysis of 48 studies measuring beliefs about diabetes and their relationship with adherence to self-care behaviours found beliefs significantly associated with higher adherence to self-care included the belief that medication was effective, higher perceived severity of diabetes, perceived control over self-care, and belief that adherence to self-care has benefits [63]. In the same study, beliefs significantly associated with lower adherence included that there is no need for medication when blood glucose levels are normal, worry about effects of medications, and low confidence in controlling diabetes [63]. These results are in line with our finding. Survey-collected study data indicated that a third of participants reported experiencing a diabetic complication during TB treatment (Table 2). Participants in this study expressed a key motivation to use DM medications was a sense of vulnerability to diabetic complications and a belief that glucose-lowering medications were effective, while barriers included beliefs that the medications were not necessary, effective, or caused harm.

The perceived severity of DM and its complications may influence whether an individual engages in self-care practices [39, 64–66]. Previous studies have found that people who perceive their diabetes as more severe had better adherence to diabetic self-care practices [39, 67–69]. As diabetes is a progressive disease, those who have had diabetes for longer likely perceive the severity of their disease differently than recently-diagnosed persons [67, 70]. In this study, most participants with uncontrolled glycaemia more often discussed experiencing diabetic complications like neuropathy and retinopathy, whereas few persons with controlled blood sugar mentioned perceiving any symptoms of hyperglycaemia. Survey-collected data was inconclusive: approximately a third of the 31 participants self-reported a diabetic complication during TB treatment, with equal proportions between those with controlled versus uncontrolled blood glucose control (Table 2).

The results of our study were in line with other studies of diabetic populations, which have found that persons with diabetes postpone lifestyle changes or use of medication until experiencing diabetic complications [70]. However, there are currently no studies which examine how acute TB infection may affect perception of diabetes severity and motivation to manage diabetes. We found that participants with uncontrolled glycaemia more often recounted that experiencing acute illness compelled them to manage their diabetes through lifestyle changes or medications.

### Self-care agency

This study identified that a potential barrier to providing DM resources through TB-DOTS centres was the perception that DM management was beyond the remit of TB-DOTS providers. Often subjects sought information about DM and its management from the internet and their community, especially when they perceived the resources available through TB-DOTS as inadequate. A qualitative study of barriers to diabetic self-management among First Nations people in Alberta yielded a similar finding [49]. In our study, a few participants thought DM services could not be accessed through their TB-DOTS centre. Our study supports that where DM screening and medications are available, TB patients should be informed about the DM resources available through the TB-DOTS centre or its affiliated health centre.

### Access to resources

Screening of newly registered TB patients for diabetes is recommended by WHO [71], and is presently being considered for implementation in the Philippines through its Universal Health Coverage scheme. This study identified that most participants who were newly diagnosed with DM at the start of treatment had not perceived any complications of DM before, and may have not otherwise sought out consult for diabetes. Survey-collected participant data did not capture whether patients had perceived symptoms of DM prior to their TB illness (Table 2); the qualitative investigation lent critical insight to patients’ temporal perception of DM. Systematic reviews of bi-directional screening for TB and DM have similarly found screening TB patients for DM may identify a large number of new cases which might have otherwise gone undiagnosed until complications of chronic hyperglycaemia manifested [72, 73]. If health authorities in the Philippines, or countries with a similar disease burden, pursue a programme of DM screening through TB-DOTS, care should be taken to confirm diagnoses outside of the acute phase of TB treatment to avoid overdiagnosis, as symptoms of active TB disease overlap with classic symptoms of DM (e.g. unexplained weight loss and extreme fatigue), and patients starting TB treatment may experience temporarily elevated blood glucose occurring as part of a stress response to acute TB disease [15, 28, 74–76].

Consulting previously-diagnosed DM patients about their diabetes and blood glucose control at the start of TB treatment may be an important link to DM care. This study found accounts of participants with previously-diagnosed DM who had not engaged in self-care activities until experiencing ill health from their TB infection or receiving provider feedback about their HbA1c results during TB treatment. Ensuring TB-DOTS programmes are systematically linked to DM resources, and are supported by primary health clinics responsible for long-term management of DM patients is critical. Global guidelines state that while DM care and treatment should be delivered by a DM clinic, during the first two weeks to two months of TB treatment, TB-DM patients should not be referred to DM clinics to avoid exposing people in DM clinics to potential TB transmission [77].

This study found that financial constraints, especially the loss of employment due to TB infection and the purchase of DM medications out-of-pocket, were a key barrier to diabetic self-management. More than half of participants in this study reported an absolute annual household income of less than 9,999 PHP (approximately €163) (Table 2). Financial constraints have also been identified as a barrier to self-care across other studies among populations with DM [49, 50, 78, 79].

Receiving free glucose-lowering medications was the most commonly mentioned enabler to self-care across all glycaemic control groups, helping participants overcome financial barriers. Glucose-lowering medications are, in principle, free for all patients through the national health insurance programme, PhilHealth, and can be accessed through health centres or their affiliated TB-DOTS unit or barangay health station. Insulin is additionally available for patients receiving TB treatment through PMDT facilities. Many newly diagnosed participants in this study were initiated medications for their DM during TB treatment, and others previously paying for medications out-of-pocket previously switched to the free medications accessed through TB-DOTS. In our study setting, all facilities had supply of at least one free glucose-lowering medication (usually metformin), and all PMDT centres offered fasting blood glucose testing and access to insulin. However, several participants purchased medications out-of-pocket, perceiving a lack of availability at their TB-DOTS centre.

### Continuity of DM management after TB treatment

Patients who complete TB treatment still need to receive long-term DM management; a concern in linking TB and DM care is that pathways for continued DM management after TB treatment may be non-existent or inaccessible due to availability, affordability or quality [71]. Participants in PMDT programmes had access to additional resources for DM management (e.g., medicines or blood glucose testing) that were not available through TB-DOTS or health centres. PMDT programmes provide a weekly allowance, as well as insulin and needles. Most participants using insulin stated they had received it through PMDT, and relied on their weekly allowance to purchase insulin or needles in the case of stock-outs. This finding is concerning as it suggests that patients, especially those with more advanced DM, may be unable to access insulin after finishing their TB treatment. TB programmes offering resources for DM management should ensure patients are linked with the local health centre at TB treatment completion to ensure continuity of DM care.

Finally, though data was examined within each glycaemic group, no clear themes emerged which explained why some participants experienced poor glycaemic control. We expected to find that persons with uncontrolled glycaemia were less aware of diabetes and its management, and did not feel motivated to engage in self-care. Instead, we found that persons with controlled blood glucose were often recently diagnosed with uncomplicated disease, and persons with uncontrolled blood glucose were aware of their disease severity and knowledgeable of and engaged in diabetes management.

### Strengths and limitations 

This study highlights subjective perspectives about diabetic management while in TB treatment across a diversity of treatment settings. This is the first qualitative study which elicits accounts of DM self-care from persons in TB treatment. Though there are other qualitative studies which explore barriers and enablers to diabetic self-management among persons in low-resource settings [49–52, 78, 79], and systematic reviews of barriers and enablers to self-management in low-and middle-income countries [18, 23, 24], none of these works consider the unique challenge of self-management of DM during TB treatment.

There were several limitations. We aimed to sample until a point of saturation was reached, relying on discussions with research assistants after each interview and review of translated transcripts to judge when no new questions or concepts were emerging from interviews. However, due to delays introduced by the COVID-19 pandemic and the small number of eligible participants enrolled with DM at the time of data collection, it was not possible to reach a clear point of saturation for all key issues. For example, additional accounts of participants from additional study sites were needed to better understand how diabetes resources were perceived and accessed. This may have impacted on content validity, and diminished the generalisability of findings. We expanded eligibility to 13 participants who were still participating in the St-ATT study, but had finished treatment for TB within the past six months. Recall bias may affect the accounts of DM self-management among these participants.

Due to the COVID-19 pandemic, 15 interviews were conducted by phone. It was challenging to establish rapport and impossible to perceive body language. Poor phone connectivity interrupted several interviews, and two participants with poor hearing needed a family member to participate in the interview on their behalf.

Finally, though qualitative interviews were conducted by Filipino research assistants who were experienced in health research, the lead author was a linguistic and cultural outsider. This created potential for the loss of information from tone and body language, and for the misinterpretation of findings. To mitigate this limitation, the PI consulted with Filipino health researchers and health workers living with diabetes while developing the interview guide to gain an understanding of how diabetes was understood in Filipino culture and support the culturally-appropriate translation of concepts into local languages. The lead author observed all in-person interviews, taking detailed field notes during interviews and debriefing directly after each interview with the research assistant to discuss interview content, clarify misunderstandings, identify gaps in the interview guide, and address problems in data collection.

## Conclusion

TB-DOTS can serve as a key point of connection to resources for diabetic management. This study found that many participants who were newly-diagnosed at the time of enrolment in TB treatment had not perceived any diabetic symptoms prior and would have likely not sought clinical consult otherwise. Additionally, experiencing acute illness from TB may incline those who had been previously diagnosed but not yet practicing diabetic self-care to be receptive to clinical consult about their DM, its management, and public resources potentially available to them. This study found that additional financial resources offered through PMDT were used to purchase more expensive diabetic supplies like insulin—it is important to bridge access to these resources following the completion of TB treatment. Diabetic screenings and consults at the time of enrolment in TB-DOTS programmes could facilitate diabetic people accessing public resources and initiating long-term self-care practices. However, this study also found glucose-lowering medications were not accessible to all participants through health centres. The Department of Health should continue to expand and strengthen its Universal Health Coverage programme to ensure all DM services are reliably accessible. Finally, TB programmes might consider monitoring and managing diabetes among groups with potentially greater needs—such as those with a previously-diagnosed or advanced disease requiring pharmaceutical intervention to reduce risk of diabetes-related catastrophic health costs during TB treatment, as many participants expressed that the financial and physical impact of TB made it difficult to engage in diabetic self-care.

### Supplementary Information

**Additional file 1.** COREQ Checklist.

**Additional file 2.** Interview guide.

## Data Availability

The data used and analysed during the current study are available from the corresponding author on reasonable request and permission of the ST-ATT Principal Investigators.

## Abbreviations

DM: Diabetes Mellitus
HbA1c: Glycosylated haemoglobin
TB: Tuberculosis
TB-DM: Tuberculosis with a diabetes mellitus comorbidity
TB-DOTS: Directly Observed Treatment for the Treatment of Tuberculosis
MDR-TB: Multidrug-resistant tuberculosis
PHP: Philippine peso
PMDT: Programmatic management of drug resistant TB
St-ATT: Starting Anti-TB Treatment cohort

## Footnotes

1 Amongst those with ≥ 2 HbA1c test results: Controlled = at least two HbA1c results < 8%, and uncontrolled =  ≥ 2 HbA1c results ≥ 8%. When there was insufficient HbA1c data, an indeterminate case type was assigned.
