# Supplementary material for: How can tuberculosis services better support patients with a diabetes co-morbidity? A mixed methods study in the Philippines
Source: BMC Health Serv Res. 2023 Sep 25;23:1027. doi: 10.1186/s12913-023-10015-7 (PMC10519082; doi:10.1186/s12913-023-10015-7)
Supplement: Supplementary file 1 — Additional file 1. COREQ Checklist. [file 12913_2023_10015_MOESM1_ESM.docx]

# Additional File 1: COREQ Checklist

**Title:** How can tuberculosis services better support patients with a diabetes co-morbidity? A qualitative study in the Philippines

**Authors:** Lauren Oliveira Hashiguchi (LOH), Sharon E Cox (SC), Tansy Edwards (TE), Mary C Castro (MC), Mishal Khan (MK) , Marco Liverani (ML)

**Fieldwork Assistants:** Serafin Malecosio (SM), Josephine Avila (JA)

| **No** | **Item** | **Description** | **Response** |
| --- | --- | --- | --- |
| **Domain 1: Research team and reflexivity** | | | |
| Personal characteristics | | | |
| 1. | Interviewer/facilitator | Which author/s conducted the interviews or focus groups? | SM, JA |
| 2. | Credentials | What were the researcher's credentials? E.g. PhD, MD | LOH (MSPH, PhD) was a PhD Candidate at the London School of Hygiene and Tropical Medicine and Nagasaki University.  SC (PhD) was a professor  Tansy Edwards (TE), Mary C Castro (MC),  ML (MSc, PhD) was an associate professor in health policy at the London School of Hygiene and Tropical Medicine.  MK (MA, MSc PhD) was a professor of health policy at the London School of Hygiene and Tropical Medicine.  TE (MMATH, MSc, PhD) was a associate professor in statistics and epidemiology at the London School of Hygiene and Tropical Medicine and at the Nagasaki University Graduate School of Topical Medicine and Global Health in Japan.  SC (MSc, PhD) was a Professor of Epidemiology and Nutrition at Nagasaki University Graduate School of Topical Medicine and Global Health in Japan.  MC (MD) was the Executive Director at Nutrition Center of the Philippines.  SM (MSPH, PhD Candidate) was an Assistant Professor in public health at the University of the Philippines Visayas.  JA (MA) was an Assistant Professor in Population Studies at the University of San Carlos (Philippines). |
| 3. | Occupation | What was their occupation at the time of the study? |  |
| 4. | Gender | Was the researcher male or female? | LOH, MK, SC, TE, MC, and JA are female. ML, SM are male. |
| 5. | Experience and training | What experience or training did the researcher have? | LOH and MK and all fieldwork researchers had postgraduate education in social sciences.  ML and MK are social scientist with extensive experience in qualitative methods. ML provided further training on interview techniques to LOH in preparation for fieldwork.  LOH was a PhD Candidate in global health with training in qualitative methods. To gain practical field experience in conducting qualitative interviews in the Philippines before initiating data collection, LOH observed field interviews over several days in September 2019 between local research assistants and hypertensive patients in Quezon, Philippines through the Responsive and Equitable Health Systems—Partnership on Non-Communicable Diseases (RESPOND) study.  Both research assistants had previous experience conducting qualitative interviews about health topics in a Filipino context. LOH additionally gave qualitative training to all research assistants in preparation to fieldwork, with oversight from ML and MK. |
| Relationship with participants | | | |
| 6. | Relationship established | Was a relationship established prior to study commencement? | The research team was not acquainted with study participants prior to the study. Participants were initially contacted through St-ATT research nurses, whom they interacted with on a monthly basis for extended periods of time. |
| 7. | Participant knowledge of the interviewer | What did the participants know about the researcher? e.g. personal goals, reasons for doing the research | All participants were informed about study aims and objectives (i.e. to explore barriers and facilitators to the management of diabetes within a TB context, with a view to improving health services delivery). Researchers introduced themselves to all participants, providing information about their work and employment |
| 8. | Interviewer characteristics | What characteristics were reported about the interviewer/facilitator? e.g. Bias, assumptions, reasons and interests in the research topic |  |
| **Domain 2. Study design** | | | |
| Theoretical Framework | | | |
| 9. | Methodological orientation and theory | What methodological orientation was stated to underpin the study? e.g. grounded theory, discourse analysis, ethnography, phenomenology, content analysis | As detailed in the manuscript, the study design was supported by a conceptual framework derived from the literature on self-care among people with chronic health conditions. |
| 10. | Sampling | How were participants selected? e.g. purposive, convenience, consecutive, snowball | Research sites were purposively selected using a combination of criteria, in keeping with study objectives (i.e., on the basis of blood glucose control patterns measured during TB treatment, and other key characteristics identified through analysis of patient cohort data). |
| 11. | Method of approach | How were participants approached? e.g. face-to-face, telephone, mail, email | Participants were approached face-to-face at their TB clinic. During the COVID-19 community quarantines, interviews were conducted over the phone. |
| 12. | Sample size | How many participants were in the study? | 31 in total |
| 13. | Non-participation | How many people refused to participate or dropped out? Reasons? | None of the individuals approached for interview refused to participate, though an additional participant’s interview was stopped when it became apparent that they did not consider themselves to have DM. |
| 14. | Setting of data collection | Where was the data collected? e.g. home, clinic, workplace | At TB-DOTS clinics, or over the phone |
| 15. | Presence of non-participants | Was anyone else present besides the participants and researchers? | Relatives of the interviewee were occasionally present during the interview. |
| 16. | Description of the sample | What are the important characteristics of the sample? | Key demographic data are provided in Table 1. |
| Data collection | | | |
| 17. | Interview guide | Were questions, prompts, guides provided by the authors? Was it pilot tested? | Following a literature review, input from LOH’s PhD supervisory team (ML, MK, SC, TE, CC), topic guide was validated by the research assistants and St-ATT study nurses for comprehensibility and equivalence of meaning, and was tested in mock interviews with study nurses, and in pilot interviews with St-ATT participants |
| 18. | Repeat interviews | Were repeat interviews carried out? If yes, how many? | Yes, three interviews in Cebu were repeated as they were initially conducted in Tagalog, and the participants preferred Cebuano. |
| 19. | Audio/visual recording | Did the research use audio or visual recording to collect the data? | All interviews were audio recorded |
| 20. | Field notes | Were field notes made during and/or after the interview or focus group? | Observational field notes were taken at each fieldwork site |
| 21. | Duration | What was the duration of the interviews or focus group? | The duration of interviews ranged from 30 min to 1 hour approx. The average duration was about 45 minutes. |
| 22. | Data saturation | Was data saturation discussed? | Yes, at different stages of data collection and within the discussion. |
| 23. | Transcripts returned | Were transcripts returned to participants for comment and/or correction? | No. This would have been logistically difficult and inappropriate given low literacy in the study locations. |
| **Domain 3. Analysis and findings** | | | |
| 24. | Number of data coders | How many coders coded the data? | One researcher: LOH. Findings and emerging themes were repeatedly discussed with all co-authors (ML, MK, SC, TE, MC). |
| 25. | Description of the coding tree | Did authors provide a description of the coding tree? | The overarching thematic structure was developed in advance following the conceptual framework presented. Sub-themes within each category were derived from collected data in an iterative process. |
| 26. | Were themes identified in advance or derived from the data? | Were themes identified in advance or derived from the data? |  |
| 27. | Software | What software, if applicable, was used to manage the data? | QSR NVivo version 12 |
| 28. | Participant checking | Did participants provide feedback on the findings? | No. However, preliminary findings were discussed with the research assistants (SM, JA) and co-authors. |
| Reporting | | | |
| 29. | Quotations presented | Were participant quotations presented to illustrate the themes / findings? Was each quotation identified? e.g. participant number | Yes, we included many quotations. These are identified by a unique code. |
| 30. | Data and findings consistent | Was there consistency between the data presented and the findings? | Yes |
| 31. | Clarity of major themes | Were major themes clearly presented in the findings? | Yes (see section Results) |
| 32. | Clarity of minor themes | Is there a description of diverse cases or discussion of minor themes? | Yes. See, for example, the paragraph beginning with “While there was a general trust in instructions…” |
