# Supplementary material for: How can tuberculosis services better support patients with a diabetes co-morbidity? A mixed methods study in the Philippines
Source: BMC Health Serv Res. 2023 Sep 25;23:1027. doi: 10.1186/s12913-023-10015-7 (PMC10519082; doi:10.1186/s12913-023-10015-7)
Supplement: Supplementary file 2 — Additional file 2. Interview guide. [file 12913_2023_10015_MOESM2_ESM.docx]

**Pre-interview**

- 1. **Introduction – building up to descriptive talking**
     1. Thank you so much for your time
        1. If needed, summarize introductory statements from recruitment script
        2. Tell me, how are you today?
  2. **Topic: Perception of DM service delivery**

**B1.** What do you think about your TB clinic? Do they take good care of you?

Probes (if appropriate):

Can you tell me about what you like about receiving treatment here?

What about things you dislike, or could be better?

Why are these things important to you?

**B2.** Can you tell me about the treatment you receive for your **diabetes** at this TB-DOTS clinic (e.g., medicines and medical supplies, financial support, education, blood sugar tests)?

Probes:

Can you tell me about what you like about receiving diabetes treatment here?

What about things you dislike, or could be better?

Why are these things important to you?

**B3.** How do you feel about treating your diabetes?

**B3.1.** What are some problems you face in treating your diabetes?

**B3.2.** What are some things that make it easier or more convenient for you to treat your diabetes?

**B4.** Did you ever receive diabetes treatment at another facility? Can you tell me about the diabetes treatment they give you? Do you still go there?

- *If they do not go to outside facility any longer:*

Why did you stop going to this facility for DM treatment?

- *If they continue to seek treatment at outside facility:*

Why do you prefer to go to this facility for your diabetes instead of/ in addition to your TB-DOTS facility? What do you like about receiving DM services there? Why are these things important to you?

**B5.** Has there been a time when you need information, medicine, or testing for you diabetes, but could not receive it at this/[or other usual] clinic? What did you need, and why were you not able to get it? Were you able to get these things elsewhere?

**B6.** Some people told me it is difficult to follow all the advice about diabetes treatment from nurses, what do you think?

- 1. **Topic: Knowledge and feelings of diabetes**

**C1.** How is your diabetes, or high blood sugar?

**C2.** Could you tell me something about the day you were diagnosed with diabetes? Do you have any memories you can share…?

- 1. **Topic: Attitudes, and practices around diabetes**

**D1.** Does diabetes affect your life? Does diabetes have an impact on your relations with family and friends, daily activities, job?

- 1. **Final**

**E1.** What is your greatest priority with your health right now?

**E2.** Of all the things we’ve talked about today – or maybe some topics I missed – what should I pay most attention to?
